# Prelabour Rupture of Membranes: Mode of Delivery and Outcome

**DOI:** 10.3889/oamjms.2015.037

**Published:** 2015-04-24

**Authors:** Vlora Ademi Ibishi, Rozalinda Dusan Isjanovska

**Affiliations:** 1 *University Clinical Center of Kosovo - Obstetrics and Gynecology Clinic, Prishtina, Kosovo*; 2 *Institute for Epidemiology and Medical Biostatistics, Medical Faculty, Ss. Cyril and Methodius University of Skopje, Skopje, Republic of Macedonia*

**Keywords:** prelabour rupture of membrane (PROM), latency period, mode of delivery, neonatal outcome, maternal complications

## Abstract

**BACKGROUND:**

Pre-labour Rupture of Membranes (PROM) is an important cause of maternal and fetal morbidity and increased rate of cesarean section delivery.

**AIM:**

The aim of this study is to investigate the clinical characteristics, PROM-delivery interval, mode of delivery, and early maternal neonatal outcome among pregnant patients presenting with pre-labour rupture of membranes.

**MATERIAL AND METHODS:**

This prospective case control study is implemented at the Obstetric and Gynecology Clinic of the University Clinical Center of Kosovo. The study included 100 pregnant patients presenting with prelabour rupture of membranes of which 63 were primigravida and 37 patients were multigravida.

**RESULTS:**

The incidence of cesarean section in this study is 28 % and the most common indications for cesarean delivery were fetal distress, malpresentation, cephalopelvic disproportion, and failed induction. The most common maternal complications in this study are chorioamnionitis, retained placenta and postpartum hemorrhage. Neonatal infectious morbidity was present in 16 % of cases.

**CONCLUSION:**

PROM is a significant issue for obstetricians and an important cause of maternal and neonatal morbidity and increased rate of cesarean section delivery.

## Introduction

Spontaneous rupture of membranes (ROM) is a normal component of labour and delivery [1], but the pre-labour rupture of membranes (PROM) is not. PROM refers to rupture of the membranes prior to the onset of labor and prior to the onset of clinically apparent labour contractions [2]. PROM can occur at any gestational age and is classified as “preterm PROM” if the event occurs before 37 weeks of gestation or “term PROM” if the event occurs after 37 weeks of gestation [3-5]. A number of issues are associated with PROM including infection, anatomic and pregnancy-related factors [6]. PROM is related to number of adverse maternal and neonatal outcomes. The most frequent maternal consequences associated with PROM are chorioamnionitis, endomyometritis, wound infection, pelvic abscess, bacteremia and postpartum haemorrhage [7, 8]. One of the most serious consequences of PROM-related maternal infection is Early Onset Neonatal Infection (EONI). EONI is often acquired prenatally in pregnancies with PROM and is associated with increased neonatal morbidity and mortality. 9 Another adverse outcome of prelabour rupture of membranes is the increased use of operative procedures which can increase the likelihood of a cesarean section.

The aim of this study was to investigate the clinical characteristics, PROM-delivery interval, mode of delivery, and early maternal neonatal outcome among pregnant patients presenting with pre-labour rupture of membranes in Republic of Kosovo.

## Material and Methods

This prospective case control study was implemented at the Obstetric and Gynecology clinic of the University Clinical Center of Kosova and included 115 pregnant patients. This study was submitted and approved by the Ethical Review Committee of the University Clinical Center of Kosova and is in adherence to the laws and regulations of the country in which the research was conducted.

Inclusion criteria for participant eligibility included that women were between 28-41 weeks of gestational age, not on either antibiotic or corticosteroid treatment, and experienced pre-labour rupture of amniotic membranes were assessed for eligibility. Women were excluded (N = 15) who had hypertensive disorders, diabetes mellitus, fetal malformations or other co-morbidities. The study was thus conducted on 100 women (and their newborns, N = 100) who met study eligibility criteria. Study participants were divided into two groups according to their gestational age at the time of rupture of membranes: 1) Term PROM with gestational age ≥ 37 weeks of gestation (69 cases), and 2) Pre-term PROM with gestational age 28-36 weeks+6 days (31 cases).

After implementing informed consent process a detailed patient history and examination was performed and confirmation of the diagnosis of rupture of membranes was documented. The documenting of the rupture of membranes was done by sterile speculum examination confirming the pooling of amniotic fluid in the posterior vaginal fornix or/and direct visualization of fluid leakage from the cervical canal. A questionnaire and evaluation form was used to collect data at admission. Demographic data, clinical characteristics and data covering PROM –delivery interval, mode of delivery and maternal neonatal outcome were recorded and compared between the two groups. Total hospital stay was also recorded and analyzed.

## Results

A total of 100 patients with PROM were analyzed in this study. Demographic data, obstetrical risk factors for PROM respectively previous PROM, previous abortions and smoking in pregnancy are presented in Table 1. A majority of the study participants were young (between 20-29 years), unemployed (89%), were in the socioeconomic middle class (73%), and completed up to secondary education (72%). Twenty-two percent were smokers and 29% had previous abortions. Out of 37 multiparous patients, 59% (N=22) of them had experienced previous PROM.

**Table 1:** Demographic data of the patients.

Variable	n 100 (%)
Maternal age	
15-19 years	3 (3%)
20-29 years	65 (65%)
30-39 years	32 (32%)
Socio economic status	
Low	18 (18 %)
Middle	73 (73%)
High	9 (9%)
Education	
Elementary	26 (26%)
Secondary	48 (48%)
University	26 (26%)
Employment status	
no	89 (89 %)
yes	11 (11%)
Smoking	
Yes	22 (22%)
No	78 (78%)
Previous abortions	
Yes	29 (29%)
No	71 (71%)
Previous PROM (N=37)	
Yes	22 (59%)
No	15 (41%)
No previous birth	63 (63%)

The clinical characteristics of investigated patients with PROM are presented in Table 2. Of the 100 study participants, 63% (N = 63) were primigravida and 37% (N = 37) were multigravida. The mean interval between the rupture of the membranes and the onset of labour was 18.4 hours for the Term PROM group and 26.3 hours for the Pre-term PROM group. Statistical analysis reports the duration of hospitalization significantly longer (9.3 ± 7.8) in the Pre-term PROM group compared to the term PROM group (3.5 ± 3.0).

**Table 2:** Clinical Characteristics of the Term and Preterm PROM groups.

Variable	Total n=100	Group I	Group II	
		Term PROM	Pre-term PROM	
		N = 69	N = 31	
Parity				
Primiparous n (%)	63 - 63.0%	46 - 66.7%	17 - 54.8%	p= 0.257
Multiparous n (%)	37 - 37.0%	23 - 32.3%	14 - 45.2%	
Latency period/hours (Mean SD)	20.86 ± 12.6	18.4 ± 9.0	26.3 ± 17.4	p=0.132
Hospitalization /Days	5.3 ± 5.67	3.5 ± 3.0	9.3 ± 7.8	P<0.001
Gestational week at birth (Mean SD)	37.5 ± 2.5245	38.9 ± 0.99	34.4 ± 1.94	
Mean of neonatal weight (gram)	3040.70 ± 628.17	3332 ± 377.15	2392 ± 591.65	

Mode of delivery and maternal- neonatal outcomes are presented in Table 3. Induced vaginal delivery was most common (38%, N = 38), while spontaneous vaginal delivery rate was 34% (34 cases).

**Table 3:** Mode of delivery and neonatal- maternal outcome in the studied groups.

Variable	Total n=100	Group I Term PROM n=69	Group II Preterm PROM n=31	p value
Mode of delivery				
Spontaneous	34-34.0%	22-31.9	12-38.7	p=0.084
Induced	38-38.0	31-44.9	7-22.6	
Cesarean Section	28-28.0	16-23.2	12-38.7	
Neonatal infectious morbidity	Yes 16 (16.0 %) No-84 (84.0)	6-8.7% of total-69 6-37.5% of 16 No-63	10-32.2 of total-31 10 – 62.5% of 16 No-21	p=0.003
Maternal complications	Yes-8 (8.0 %) No-92(92.0)	5- 7.2% of total-69 5 – 62.5% of 8 No-64	3-9.7% of total-31 3–37.5% of 8 No-29	p=0.680

The cesarean section rate was 28 % (N = 28). Comparison of the cesarean section rate between the Term Group and the Preterm Group did not show any significant statistical difference. Neonatal infectious morbidity was present in 16% (N = 16) of cases. Out of the N = 16 neonatal infection cases, 6 of them were in the Term Group and 10 were in the Pre-Term Group, which is a statistically significant difference (p = 0.003). Main maternal complications were chorioamnionitis, postpartum haemorrhage, and retained placenta. Both Term and Pre-Term groups showed similar rates of maternal complications (p = 0.680).

**Figure 1:**
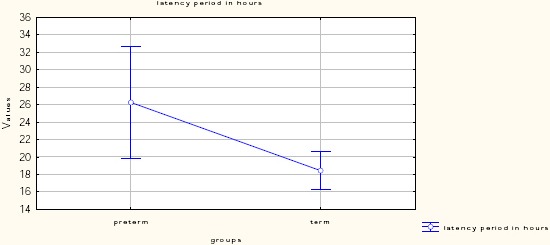
*Latency period in hours*.

**Figure 2:**
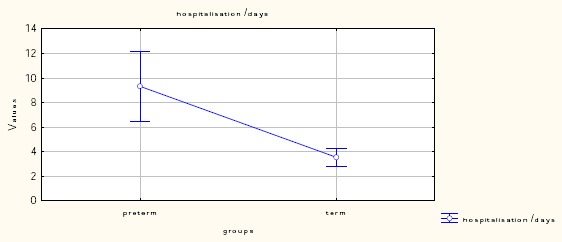
*Hospitalization/Days*.

## Discussion

Pre-labour rupture of membranes remains one of the important problems in obstetric practice. The etiology of PROM is multi-factorial and in some cases yet unclear. Infection is one of the exogenous etiologic factors thought preventable in some cases with proper antenatal screening and treating of genito-urinary infections. Management of pregnancies with pre-labour rupture of membranes varies depending on gestational age and obstetric status. Recent clinical trials support immediate labour induction in pregnant woman with PROM at term in absence of other maternal and fetal contraindications. Management of Pre-term PROM is more complex and carries risk of prematurity and other related fetal complications.

Maternal complications, increased rate of operative delivery, increased rate of neonatal morbidity and mortality in pregnancies with PROM are reported in the literature, and PROM is also more frequent in developing countries. This study was conducted with the aim to evaluate these characteristics, mode of delivery and outcome of pregnancies with pre-labour rupture of membranes in a local setting. This study included one hundred patients of which 63 were primigravida and 37 patients were multigravida. Our findings suggest higher incidence of PROM in the primiparous than in the multiparous patients. The participants in this study had a wide variation in age, from 17 years to 37 years.

The literature reports that PROM is associated with an increased risk of cesarean delivery [10]. The results from this study report a cesarean section rate 28 %, with no significant statistical difference between Term and Preterm PROM groups. This 28% with cesarean section reported in this study is similar to percentages found in other studies. In a study with 536 cases, in Iran, Eslamian, et al (2002), reports a study the cesarean section in cases with PROM was 28.08 % [11], Chakraborty et al (2013) reported a Cesarean Section rate of 26.6 % among pregnancies with prelabour rupture of membranes in a study conducted in West Bengal [12]. Kunze et al (2011), who conducted a study with 1026 cases with PROM in their study about intrapartum management of premature rupture of membranes has reported a cesarean section rate of 27 % [13].

In this study, fetal distress, malpresentation, cephalopelvic disproportion, and failed induction were the most common indications for cesarean delivery. Two of the 28 cesarean sections performed in this study were done so on the request of the mother. Of the studied cases maternal complications were present in 8% of cases. The most common maternal complications were chorioamnionitis, retained placenta and postpartum hemorrhage. There was no statistically significant different in maternal complication between the Term and Pre-Term PROM groups.

Neonatal infectious morbidity was present in 16 cases. Of these cases, observed neonatal infectious morbidity was statistically significantly higher (p = 0.003) in the Pre-term PROM group (62.5 compared to Term PROM Group (37.5 %). In addition to the risk of prematurity the preterm infants of pregnancies with pre-labour rupture of membranes are at higher risk of infectious morbidity.

In conclusion, pre-labour rupture of membranes remains an important cause of maternal and fetal morbidity and increased rate of cesarean section delivery. Postpartum haemorrhage, retained placenta, and chorioamnionitis are the most common maternal complications. Neonatal infection related to PROM is also an important factor of neonatal morbidity especially in pre term born infants. Fetal distress, malpresentations, cephalopelvic dispro-portion and failed induction are the most common indications for cesarean section delivery in pregnancies with pre-labour rupture of membranes. The results in this study indicate PROM is a significant issue for maternal and neonatal health in Kosovo. Future studies are warranted to improve maternal-neonatal outcomes.
